# Microbiome and Metabolome Analyses of Milk From Dairy Cows With Subclinical *Streptococcus agalactiae* Mastitis—Potential Biomarkers

**DOI:** 10.3389/fmicb.2019.02547

**Published:** 2019-11-06

**Authors:** Jinjin Tong, Hua Zhang, Yonghong Zhang, Benhai Xiong, Linshu Jiang

**Affiliations:** ^1^Beijing Key Laboratory for Dairy Cow Nutrition, Beijing University of Agriculture, Beijing, China; ^2^State Key Laboratory of Animal Nutrition, Institute of Animal Science, Chinese Academy of Agricultural Sciences, Beijing, China

**Keywords:** milk microbiome, metabolomics, *Streptococcus agalactiae*, mastitis, dairy cows

## Abstract

The microbial ecosystem in the udders of dairy cows directly influences the flavor and quality of milk. However, to our knowledge, no published research has analyzed the complex relationship between the udder microbiome and its associated metabolism in animals with subclinical mastitis. We identified the bacterial species and measured relative population numbers in the milk of cows with subclinical *Streptococcus agalactiae* mastitis (GBS) and compared this information to that from the milk of healthy cows. Metabolite profiles were determined to investigate correlations between the milk microbiota and metabolic factors in healthy vs. GBS dairy cows. Six milk samples from GBS cows and six from healthy cows were subjected to 16S rRNA gene sequencing to identify the microbial species using a MiSeq high-throughput sequencing apparatus. The metabolites present in the milk were identified by gas chromatography time-of-flight mass spectrometry. Both principal component analysis and orthogonal partial least squares discriminant analysis indicated that the metabolites were well-separated from each other in the milk samples from the two groups. GBS dramatically altered microbial diversity, and the GBS group had significantly fewer *Proteobacteria, Actinobacteria*, and *Acidobacteria* than the CON group, with greater relative abundance of *Firmicutes* (*p* < 0.01). Several bacterial genera, such as *Streptococcus*, were significantly more abundant in milk from the GBS group than in milk from the CON group, and there was a tendency for greater abundance of *Turicibacter* (*p* = 0.07) and *Enterococcus* spp. (*p* = 0.07) in the GBS group. The levels of five milk metabolites were significantly higher in the GBS group than in the CON group: phenylpyruvic acid, the homogentisic acid: 4-hydroxyphenylpyruvic acid ratio, the xanthine: guanine ratio, uridine and glycerol. Metabolic pathway analysis of the different metabolites revealed that the following were enriched in both groups: galactose metabolism; pentose and glucuronate interconversion; starch and sucrose metabolism; alanine, aspartate and glutamate metabolism; arginine biosynthesis; citrate cycle (TCA cycle); D-glutamine and D-glutamate metabolism; and the neomycin, kanamycin, and gentamicin biosynthesis pathways. Several typical metabolites were highly correlated with specific ruminal bacteria, such as *Streptococcaceae, Lachnospiraceae, Lactobacillaceae* and *Corynebacteriaceae*, demonstrating the functional correlations between the milk microbiome and associated metabolites. These findings revealed that the milk microbiota and metabolite profiles were significantly different between the two groups of cows, raising the question of whether the microbiota associated with the bovine mammary gland could be related to mammary gland health. There was also a relationship between milk quality and the presence of spoilage bacteria. Other bacterial taxa should be investigated, as related information may provide insights into how perturbations in milk metabolomics profiles relate to differences in milk synthesis between healthy cows and those with subclinical mastitis.

## Introduction

Mastitis accompanied by an increase in somatic cell count (SCC) in milk is a common and costly disease in dairy cows that exhibits varying degrees of severity and reduces the quantity and quality of milk produced (Heikkilä et al., 2012; Rollin et al., 2015). Intramammary infection with *Streptococcus agalactiae* (GBS) often results in chronic subclinical mastitis (Jørgensen et al., 2016). Preventive measures, improved management, and better sanitation have reduced the number of contagious mastitis cases and have led to a change in the etiology of the disease in the last decade (Middleton et al., 2014).

Some recent studies have demonstrated the usefulness of 16S rRNA MiSeq high-throughput sequencing for revealing the effects of different intramammary antibiotic treatments on the milk microbiome (Ganda et al., 2017). This technique has been used to explore the effect of antibiotic treatment for mastitis on the microbiome in different mastitis quarters (Falentin et al., 2016) as well as differences in the microbiota in healthy udder quarters compared to cows with subclinical or clinical mastitis (Oikonomou et al., 2014). Metabolomics can be used to quantitatively measure metabolic status during lactation and the alterations in metabolites resulting from mastitis. These changes in bacterial diversity and abundance affect udder health and physiology, milk production and quality, and dairy sustainability (Flachowsky, 2006; Mansor et al., 2013; Yang et al., 2016). Metabolomics is also a useful tool for elucidating the relationships among the rumen fluid, serum, milk and urine (Sun et al., 2017); the metabolic biomarkers in different dairy animals (Sun et al., 2017); and the changes in metabolic profiles after antibiotic treatment (Junza et al., 2016). However, few studies have sought to identify and characterize unknown metabolites or degradation products in milk from cows with subclinical mastitis. The specific effect of *S. agalactiae* on the milk microbiota has also not been investigated in controlled longitudinal studies.

The objectives of the present study were to establish a set of biomarkers for determining milk microbiota diversity, to elucidate the metabolite profiles of healthy dairy cows compared to those of cows with GBS, and to explore the correlations between the milk microbiome and the milk metabolome. These goals were accomplished through 16S rRNA MiSeq high-throughput sequencing for microbiome profiling and gas chromatography time-of-flight mass spectrometry (GC-TOFMS) for metabolomics in order to compare the bacterial populations and metabolites in milk from healthy cows to those in milk from cows with GBS. Our findings will provide new insights into the dynamic metabolic mechanisms underlying GBS and enhance our understanding of how mastitis affects milk production and quality in dairy cows.

## Materials and Methods

### Animal Selection and Transportation of Milk Samples

The experimental procedures were approved by the Animal Care Committee, Beijing University of Agriculture (Beijing, China). Raw milk samples (*n* = 266) were obtained directly from commercial dairy farms in Beijing in the summer, when *S. agalactiae* numbers are elevated. The selected animals with similar parity were between 115 and 230 days in milk (DIM) and had an average SCC of 250,000 cells/ml, ranging from 110,000 to 440,000 cells/ml, measured 3 days prior to transportation to the barn. No samples were collected from cows with GBS or high SCCs, in accordance with previously published methods (Jørgensen et al., 2016). Bacteriological analyses of the 266 milk samples were performed, which revealed those cows with GBS. The experimental animals were distributed to the control [CON; healthy animals, SCC (85.13 ± 39.83 × 10^3^ cells/ml)] and GBS [SCC (329.03 ± 84.98 × 10^3^ cells/ml)] groups. There were no initial differences among the six Chinese Holstein dairy cows in each group in terms of milk yield (24.42 ± 1.68 kg/d; *p* = 0.16), DIM (154.60 ± 7.58 days; *p* = 0.71), parity (2.63 ± 0.38; *p* = 0.36), or body weight (BW; 570.25 ± 24.87 kg; *p* = 0.90). The animals were housed individually in stalls bedded with sawdust and were fed the same basal diet. The ingredients and nutrient composition of the total mixed ration (TMR) are presented in Table 1. A total of 15 ml of composite milk from each animal, with approximately equal volumes from each lactating udder quarter, was transferred to a sterile plastic bottle (Corning, Inc., Corning, NY, United States) and kept on ice until transport to the laboratory and storage at −80°C for further analysis.

**TABLE 1:** Ingredients and nutrient composition of the basal diet.

**Ingredient**	**Content, % of DM**
Alfalfa hay	6.90
Corn silage	46.32
Oat grass	2.40
Ground corn	9.88
Soybean meal	5.10
Steam-flaked corn	4.40
DDGS^1^	4.40
Corn bran	3.70
Extruded soybean	3.00
Barley	2.66
Wheat bran	2.66
Sodium cyclamate	2.40
Oats	1.50
Canola meal	1.07
Cottonseed meal	1.07
MAGALAC^2^	0.90
NaHCO_3_	0.59
Limestone	0.48
NaCl	0.27
Premix^3^	0.30
**Chemical composition,% of DM^4^**	
CP	17.4
NDF	31.1
ADF	16.6
Ether extract	5.00
Ca	0.78
P	0.44
NE_L_, Mcal/kg	1.76

*^1^DDGS = dried distillers grain with solubles. ^2^Church and Dwight, Co., Inc., Princeton, NJ, United States. ^3^Formulated to provide (per kg of DM) 4,560 mg of Cu, 3,000 mg of Fe, 12,100 mg of Zn, 4,590 mg of Mn, 60 mg of Co, 200 mg of Se, 270 mg of I, 10,000 IU of vitamin E, 450,000 IU of vitamin D, 2,000,000 IU of vitamin A, and 3,000 mg of nicotinic acid. ^4^Chemical composition based on chemical analysis of the total mixed ration (TMR), as described.*

### Bacteriological Analyses of Milk Samples

For the isolation and detection of *S. agalactiae*, all raw milk samples were first processed as described by Jørgensen et al. (2016) and Gao et al. (2017). Briefly, the milk samples were shaken, and 100 μl aliquots were removed under sterile conditions, streaked onto 5% rabbit blood agar and incubated at 37°C for 24 h. Colony morphology was observed, and several colonies were picked for Gram staining and microscopic examination. Biochemical profiling was conducted by testing for catalase activity and lactose, esculin, mannitol, salicin, raffinose, sorbitol, monosaccharide, xylose, and cellobiose fermentation. In addition, isolates were confirmed as *S. agalactiae* using the slide agglutination test for Lancefield GBS (Oxoid). The milk bacteriological analysis results are shown in Supplementary Table 1.

DNA was extracted from the milk bacteria using an InstaGene Matrix DNA Extraction Kit (Bio-Rad Laboratories, Hercules, CA, United States) according to the manufacturer’s instructions. *S. agalactiae* were then identified by PCR (Bio-Rad Laboratories) amplification of the 16S rRNA locus of the DNA. The primers were synthesized by Sangon Biotech, Co., Ltd. (Shanghai, China). A negative control (a sample without genomic DNA) and a positive control (a sample containing DNA of *S. agalactiae*, CVCC 3940, China Institute of Veterinary Drug Control, Beijing, China) were included in all PCR assays. The following primers were used: 27F: 5′-AGAGTTTGATCCTGGCTCAG-3′ and 1492R: 5′-GGTTACCTTGTTACGACTT-3′ (Saffary et al., 2002). The amplification programs were performed as described by Rubens et al. (1991). The sequence data of the isolated *S. agalactiae* strains were analyzed using the Basic Local Alignment Search Tool (BLAST) program available from the National Center for Biotechnology Information (NCBI). During universal 16S rRNA fragment sequence analysis, the cut-off level for pairwise comparisons was 99%.

### Microbial DNA Extraction and Sequencing

Microbial DNA was extracted from the milk samples using a Power Soil DNA Isolation Kit (Qiagen, Crawley, United Kingdom) according to the manufacturer’s protocols. The yield and purity of the extracted DNA were assessed with a NanoDrop 1000 spectrophotometer (Nanodrop Technologies, Wilmington, DE, United States), and DNA was stored at −80°C until use. The V1–V2 region of the bacterial 16S rRNA gene was amplified with a common primer pair (forward, 5′-CGTATCGCCT-CCCTCGCGCCATCAG-3′; reverse, 5′-CTA-TGCGCCTTGCCAGCCCGCTCAG-3′) combined with adapter sequences and barcode sequences, as previously described (Oikonomou et al., 2014). PCR amplification was performed in a total volume of 50 μl containing 10 μl of buffer, 0.2 μl of Q5 high-fidelity DNA polymerase, 10 μl of high-GC enhancer, 1 μl of dNTPs, 10 μM of each primer and 60 ng of genomic DNA. The thermocycling conditions were as follows: initial denaturation at 95°C for 5 min, followed by 15 cycles of 95°C for 1 min, 50°C for 1 min and 72°C for 1 min and a final extension at 72°C for 7 min. The products from the first round of PCR were purified through VAHTS^TM^ DNA Clean Beads. A second round of PCR was then performed in a 40 μl reaction containing 20 μl of 2 × Phusion HF MM, 8 μl of ddH_2_O, 10 μM of each primer and 10 μl of PCR products from the first step. The thermocycling conditions were as follows: initial denaturation at 98°C for 30 s, followed by 10 cycles of 98°C for 10 s, 65°C for 30 s, and 72°C for 30 s and a final extension at 72°C for 5 min. Samples with a bright main band of approximately 450 bp were chosen and mixed in equal concentration ratios as previously described (Li et al., 2018). The mixture of PCR products was purified using a GeneJET Gel Extraction Kit (Thermo Fisher Scientific, Waltham, MA, United States). The sequencing libraries were validated using an Agilent 2100 Bioanalyzer (Agilent Technologies, Palo Alto, CA, United States) and quantified with a Qubit 2.0 fluorometer (Thermo Fisher). Finally, high-throughput sequencing analysis of bacterial rRNA genes was performed on the purified pooled samples using the Illumina HiSeq 2500 platform (2 × 250 paired ends) at Biomarker Technologies Corporation, Beijing, China.

### Milk Microbiota Bioinformatic Analyses

Bioinformatics analysis was performed using both FLASH version 1.2.11 and Quantitative Insights Into Microbial Ecology (QIIME) version 1.9.1. These programs were considered to give similar results, as previously reported (Li et al., 2018). Briefly, the sequences were classified into taxa with BLAST searches in the Ribosomal Database Project (RDP) database at a 97% similarity threshold. Operational taxonomic units (OTUs) with counts greater than three in at least one of the samples were retained for further analysis. The selected OTUs were normalized to the relative abundance for each sample. The diversity of the microbial communities was estimated by using the R package phyloseq on the online BioCloud Platform^1^. Within-sample diversity (alpha diversity) was assessed through the Shannon diversity index calculated in a randomly selected subset of the OTU database. Between-sample microbial diversity (beta diversity) was assessed through phylogenetic-based weighted UniFrac distances (Catherine and Rob, 2005). For a deeper analysis of the diversity of the major evolutionary clades in the ruminal microbiota, the OTUs were filtered to acquire those with a relative abundance of at least 1% in at least one sample.

### Preparation of Milk Samples for Metabolomic Analysis

Milk samples were analyzed for specific components using an automated ultrasonic milk composition analyzer (Bentley Instruments, Chaska, MN, United States) and a method similar to our previously published procedure (Wang et al., 2017). The untargeted metabolic profiling analysis was performed by means of GC-TOFMS, and sample preparation was performed as previously described (Yan et al., 2012). The data were analyzed using an XploreMET v3.0 system (Metabo-Profile, Shanghai, China) (Chen W. L. et al., 2016). MetaboAnalyst was used to analyze range/scale data and to perform principal component analysis (PCA; De Barros et al., 2010) and KEGG pathway analysis of the altered metabolites^2^.

### Multivariate Statistical Analysis

The data on milk yield, milk composition and alpha diversity indices were analyzed using the PROC MIXED program in SAS 9.4 (SAS Institute, Inc., Cary, NC, United States), as shown in the following model: Yij = μ + Ti + ej, where Yij is the dependent variable, μ is the overall mean, Ti is the effect of treatment (no disease or GBS; considered fixed), and ej is the residual. *p* < 0.05 was considered statistically significant, and a trend was indicated by *p* < 0.10. The alpha diversity indices are presented as the mean ± SD. PCA and orthogonal partial least-squares-discriminant analysis (OPLS-DA) were performed to visualize the metabolic alterations between the experimental groups after mean centering and unit variance scaling. Variables with variable importance in the projection (VIP) values exceeding 1.0 were considered relevant for group discrimination. In this study, the OPLS-DA model was validated with sevenfold permutation tests. Significant differences in metabolites between groups were assessed using Wilcoxon rank-sum tests. A correlation matrix between metabolites and ruminal bacterial species was generated using Spearman correlation coefficients and visualized by using the R language.

### Nucleotide Sequence Accession Number

All raw sequences were submitted to the NCBI Sequence Read Archive (SRA^3^) under accession number SRP192494.

## Results

### Milk Yield and Milk Composition

The milk yield, percentage of milk fat and protein, and energy-corrected milk (ECM) content showed no difference between the two groups (Table 2). However, the yield of lactose was lower (*p* = 0.02) in the cows with GBS than in the CON cows. The percentages of lactose (*p* = 0.05) and fat (*p* = 0.08) tended to be lower in the GBS group than in CON cows. The milk from the GBS group also showed a significantly higher SCC than milk from the CON group (*p* = 0.01). Although subclinical mastitis did not significantly affect the milk yield, the percentages of lactose and fat were lower in the GBS group, indicating that the quality of the milk may have been reduced. Whether the metabolite alterations in milk from GBS cows are associated with diminished milk quality needs to be investigated further.

**TABLE 2:** Comparisons of milk yield and composition between cows with subclinical *S. agalactiae* mastitis (GBS) and healthy cows (CON).

**Yield and composition**	**CON**	**GBS**	**SEM**	***p-*value**
Yield Milk yield (kg/day)	24.42	21.78	0.679	0.16
ECM^1^ (kg/day)	27.99	23.87	1.004	0.11
Lactose (kg/day)	1.23^a^	1.03^b^	0.044	0.02
Fat (kg/day)	1.00	0.83	0.042	0.11
Protein (kg/day)	0.87	0.75	0.035	0.18
**Composition**				
Lactose (%)	5.05^a^	4.72^ab^	0.412	0.05
Fat (%)	4.11	3.82	0.105	0.08
Protein (%)	3.58	3.44	0.230	0.34
Fat: protein	1.15	1.13	0.124	0.86
SCC (× 10^4^ cells/ml)	85.13^b^	329.03^a^	62.41	0.01

*^1^ECM (kg/d) = 0.3246 × milk yield (kg/d) + 13.86 × fat yield (kg/d) + 7.04 × protein yield (kg/d) (Orth, 1992). ^*a*,*b*^For each variable, data are different if they do not share a common letter.*

### Diversity and Abundance of Microbial Communities

**FIGURE 1:**
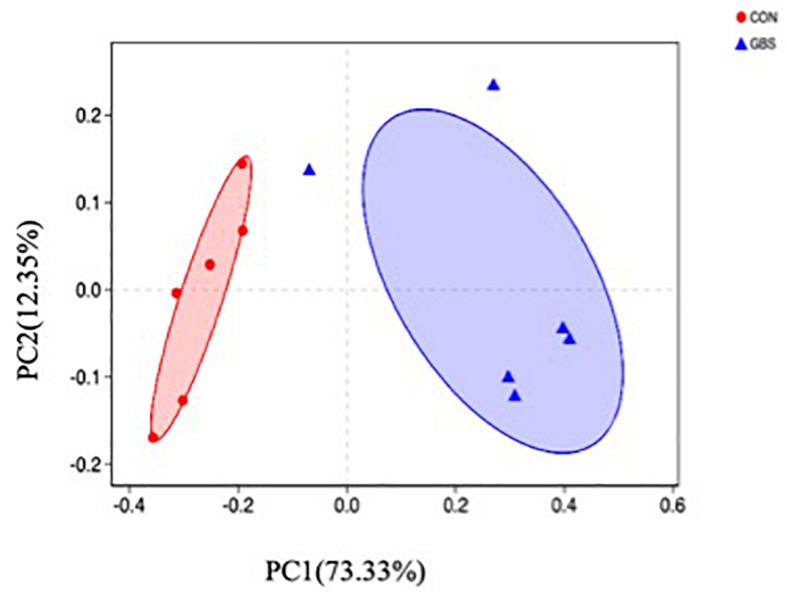
Principal coordinate analysis (PCoA) of bacterial communities in milk from healthy dairy cows (CON) and cows with subclinical *Streptococcus agalactiae* mastitis (GBS), *n* = 6.

A total of 910,984 merged sequences passed quality control, and 802,166 high-quality sequences for 12 samples were available for downstream analysis. The samples had an average length of 422 bp, and there was > 99% depth coverage, which indicated that the data volume was reasonable and could reflect changes in most bacterial flora. The alpha diversity indices of the bacterial communities in the two groups are shown in Table 3. The ACE and Chao 1 estimates of richness were not different between the two groups (*p* > 0.05). The Shannon index revealed that the diversity of microbial communities in the GBS group was significantly lower than that in the CON group (*p* = 0.05). The Shannon index and the Simpson index both show bacterial diversity. However, these values can vary depending on whether the method of calculation is based on abundance or biomass. Therefore, no significant difference was found between groups for the other alpha diversity indices. Principal coordinate analysis (PCoA) was performed to reveal distinct microbiota between the two groups, and the weighted UniFrac metrics were also determined (Figure 1). PCoA showed that principal coordinates 1 and 2 accounted for 73.33 and 12.35% of the total variance, respectively. This indicated that the microbiota in milk from cows with GBS and CON cows were markedly distinct.

**TABLE 3:** Alpha diversity indices of milk bacteria.

**Item**	**CON**	**GBS**	***p*-value**
ACE	1034.8 ± 218.8	1078.6 ± 313.03	0.80
Chao	1046.9 ± 216.9	1072.1 ± 319.89	0.88
Simpson	0.14 ± 0.05	0.27 ± 0.19	0.35
Shannon	4.12 ± 0.46	2.83 ± 1.23	0.05
Coverage	0.99 ± 0.001	0.99 ± 0.000	0.28

**FIGURE 2:**
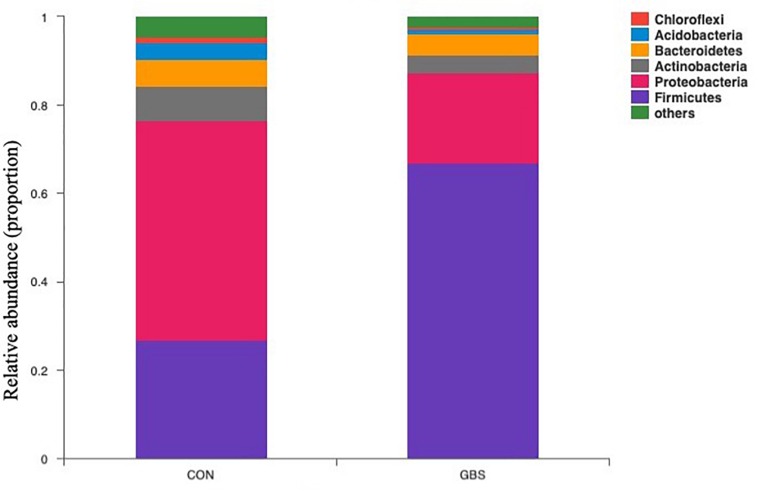
Composition of the predominant bacterial phyla identified in milk samples from healthy cows (CON) and cows with *S. agalactiae* mastitis (GBS), *n* = 6.

**FIGURE 3:**
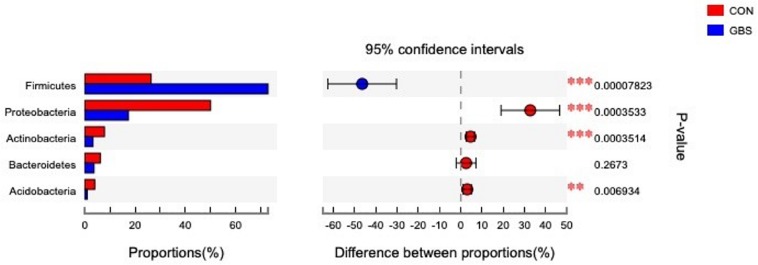
Differences between the relative abundances of five predominant bacterial phyla in milk samples from healthy cows (CON) and cows with *S. agalactiae* mastitis (GBS), *n* = 6. The extended error bar plot was generated using STAMP software. Welch’s two-sided test was used, and Welch’s inverted test was 0.95.

**FIGURE 4:**
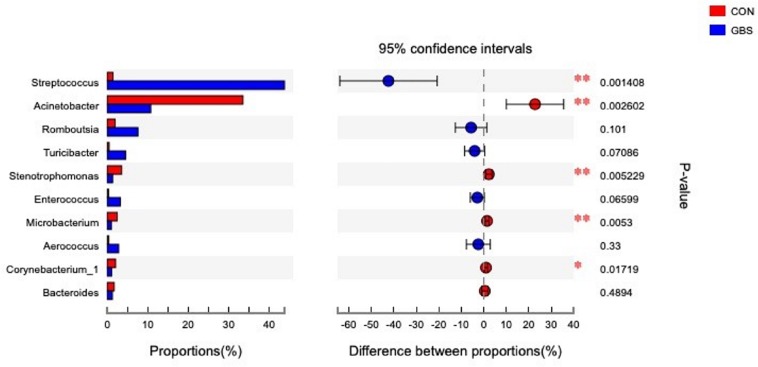
Differences between the relative abundance of the 10 predominant bacterial genera in milk samples from healthy cows (CON) and cows with *S. agalactiae* mastitis (GBS), *n* = 6. The extended error bar plot was generated using STAMP software. Welch’s two-sided test was used, and Welch’s inverted test was 0.95.

Fifty-four bacterial phyla were identified among all the samples. *Firmicutes, Proteobacteria*, and *Actinobacteria* were the three predominant phyla, representing 51.45, 32.48, and 5.34% of the total sequences, respectively (Figure 2). The remaining phyla represented less than 1% of all the sequences. We found that the GBS group had a significantly lower relative abundance of *Proteobacteria, Actinobacteria*, and *Acidobacteria* and higher relative abundance of *Firmicutes* at the phylum level than did the CON group (*p* < 0.01; Figure 3). The 10 predominant genera were identified: *Streptococcus* (24.22%), *Acinetobacter* (21.37%), *Romboutsia* (4.99%), *Turicibacter* (2.64%), *Stenotrophomonas* (2.33%), *Enterococcus* (1.86%), *Microbacterium* (1.66%), *Aerococcus* (1.60%), *Corynebacterium-1* (1.49%), and *Bacteroides* (1.44%). At the genus level, taxa with a relative abundance of 1% in at least one sample were further analyzed, and the relevant genera are presented in Figure 4. Compared to the CON group, the GBS group had a significantly increased relative abundance of *Streptococcus* and tended to show increased abundances of *Turicibacter* (*p* = 0.07) and *Enterococcus* sp. (*p* = 0.07). In contrast, the relative abundances of *Acinetobacter, Stenotrophomonas, Microbacterium*, and *Corynebacterium-1* were significantly decreased in the GBS group (*p* < 0.05; Figure 4).

### Identification and Comparison of Milk Metabolites From the Two Groups

**FIGURE 5:**
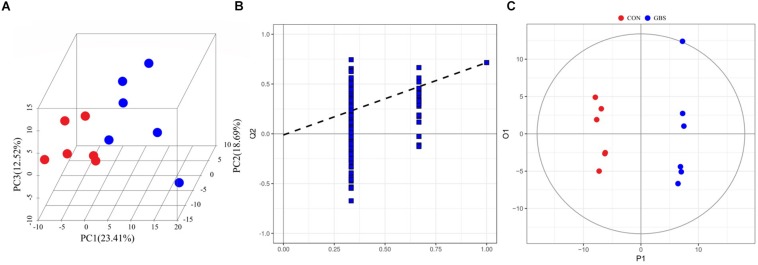
3D PCA score map **(A)**, corresponding PLS-DA validation plots **(B)**, and OPLS-DA score plots **(C)** derived from the GC-TOFMS metabolite profiles of milk from the control group (green circles) compared to the *S. agalactiae* group (blue circles).

In total, 254 measurable and reproducible metabolite signals were obtained from the milk samples. After strict quality control, 155 metabolites were identified using the *JiaLib*^TM^ metabolomics library. They consisted mainly of glycerophosphoethanolamines, fatty acids, amino acids, peptides, carbohydrates, and pyrimidines. To characterize the differences in metabolic profiles between the CON and GBS groups, PCA and OPLS-DA were conducted. The PCA score plots (Figure 5A) revealed that the milk samples from the CON group were readily separable from those of the GBS group. Principal components 1 and 2 accounted for 23.41 and 18.69% of the variation, respectively. The parameters for assessment of OPLS-DA model quality could be represented by validation plots (Figure 5B). The corresponding Q2Y value of the OPLS-DA model in milk was 0.715, and the R2Y value was 0.995. The intercept of Q2Y with a threshold < zero indicated a valid model (Q2Y = −0.012). The OPLS-DA results showed that the two groups had distinct, significantly different metabolite compositions (Figure 5C). Thus, PCA and OPLS-DA were both good indicators of the differences in metabolites in milk from control cows compared to cows with GBS.

Through a combination of the statistical analysis results and the VIP values obtained from the OPLS-DA, 22 different metabolites (*p* < 0.05 and VIP > 1) were found to be significantly different between the control group and the mastitis group (Table 4). These different metabolites were diverse, including organic acids, carbohydrates, nucleotides, amino acids, and alcohols. Milk from cows with GBS contained higher levels of pyrrole metabolites, phenylpyruvic acid, uridine, and glycerol and had a higher ratio of homogentisic acid to 4-hydroxyphenylpyruvic acid and a higher ratio of xanthine to guanine (14.29-fold higher) than did CON cows.

**TABLE 4:** Comparison of metabolite content of milk between healthy cows and *S. agalactiae*-infected cows.

**Class**	**Name**	**KEGG ID**	**VIP^a^**	***p-*value^b^**	**FC^c^**	**Tendency**
Organic Acids	Hydroxypropionic acid	C01013	1.7	0.005	0.427	↓
	Uric acid	C00366	1.7	0.012	0.491	↓
	Phenylpyruvic acid	C00166	1.7	0.014	2.628	↑
	Homogentisic acid: 4-Hydroxyphenylpyruvic acid	C00544: C01179	1.6	0.022	2.923	↑
	Oxoglutaric acid	C00026	1.2	0.025	0.397	↓
	Pyrophosphate	C00013	1.5	0.028	0.281	↓
	Quinic acid	C06746	1.5	0.031	0.497	↓
	Phosphoenolpyruvic acid	C00074	1.4	0.048	0.488	↓
Carbohydrates	Gluconolactone	C00198	1.6	0.017	0.437	↓
	Allose	C01487	1.6	0.018	0.332	↓
	1,5-Anhydrosorbitol	C07326	1.6	0.015	0.334	↓
	Sorbitol	C00794	1.6	0.015	0.533	↓
	L-Arabitol	C00532	1.7	0.019	0.483	↓
	Glucose 1-phosphate	C00103	1.7	0.019	0.162	↓
	D-Mannose	C00159	1.5	0.037	0.353	↓
	Glucose 6-phosphate	C00092	1.4	0.048	0.341	↓
Nucleotides	Uracil: uridine	C00106: C00299	1.7	0.018	0.2	↓
	Xanthine: guanine	C00385: C00242	1.5	0.038	14.29	↑
	Guanine	C00242	1.5	0.045	0.33	↓
	Uridine	C00299	1.7	0.016	4.565	↑
Amino Acids	L-Asparagine	C00152	1.4	0.037	0.493	↓
Alcohols	Glycerol	C00116	1.5	0.043	5.139	↑

*^*a*^VIP: variable importance in the projection obtained from the OPLS model with a cut-off of 1.5. ^*b*^*p-*Values from non-parametric Wilcoxon-Mann-Whitney test. ^*c*^FC: fold change, the ratio of the mean value of the peak area obtained from the *S. agalactiae* group and the mean value of the peak area obtained from the healthy group. An FC > 1 means that the metabolite is higher in milk from *S. agalactiae*-infected cows than in milk from healthy cows.*

### Metabolic Pathways Involved in Differential Metabolite Production

**FIGURE 6:**
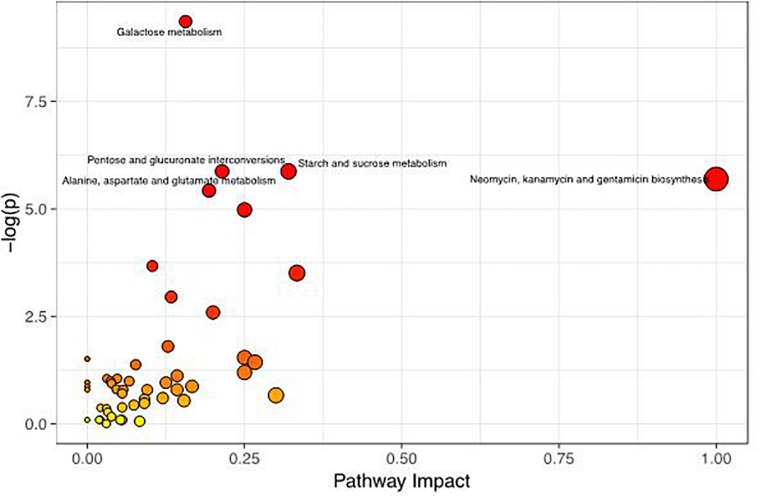
Metabolome map of changes in the common metabolites identified between healthy controls and *S. agalactiae*-infected cows. The x-axis represents the pathway impact, and the y-axis shows the pathway enrichment. Larger symbol sizes and darker colors indicate greater metabolite numbers and higher pathway impact values, respectively.

To better evaluate how multiple pathways differed between the CON group and the GBS group, a Kyoto Encyclopedia of Genes and Genomes (KEGG) functional enrichment analysis of the pathways related to the different metabolites was conducted (Table 5). The altered metabolites included organic acids, carbohydrates, nucleotides, amino acids and alcohols. Pathway topology analysis (Figure 6) with a criterion of *p* < 0.05 showed that eight main metabolic pathways were enriched between the groups, including galactose metabolism; pentose and glucuronate interconversion; starch and sucrose metabolism; alanine, aspartate, and glutamate metabolism; arginine biosynthesis; the citrate cycle (TCA, tricarboxylic acid cycle); D-glutamine and D-glutamate metabolism; and neomycin, kanamycin, and gentamicin biosynthesis pathways. The top four altered metabolic pathways between the two groups of cows were galactose metabolism, pentose and glucuronate interconversion, starch and sucrose metabolism, and the alanine, aspartate and glutamate metabolism pathways.

**TABLE 5:** Differential enrichment of metabolites in specific pathways in *S. agalactiae*-infected cows compared to healthy cows.

**Metabolic Pathway**	**Class**	**Metabolites**		***p-*value**
		**Upregulated**	**Downregulated**	
Galactose metabolism	Carbohydrate (8)	Glycerol	D-Glucose, D-galactose, D-mannose, myoinositol, sorbitol, sucrose, glucose 1-phosphate	0.000
Pentose and glucuronate interconversion	Carbohydrate (5)		D-Xylose, D-glucuronic acid, L-arabinose, glucose 1-phosphate, L-arabitol	0.003
Starch and sucrose metabolism	Carbohydrate (5)		D-Glucose, fructose 6-phosphate, sucrose, glucose 6-phosphate, glucose 1-phosphate	0.003
Alanine, aspartate and glutamate metabolism	Amino acid (6)		Fumaric acid, L-glutamic acid, L-asparagine, oxoglutaric acid, succinic acid, *N*-acetyl-L-aspartic acid	0.004
Arginine biosynthesis	Amino acid (4)		Fumaric acid, L-glutamic acid, oxoglutaric acid, citrulline	0.007
Citrate cycle (TCA cycle)	Carbohydrate (4)		Fumaric acid, oxoglutaric acid, succinic acid, phosphoenolpyruvic acid	0.025
D-Glutamine and D-glutamate metabolism	Metabolism of other amino acids (2)		L-Glutamic acid, oxoglutaric acid	0.030
Neomycin, kanamycin and gentamicin biosynthesis	Biosynthesis of other secondary metabolites (2)		D-Glucose, glucose 6-phosphate	0.003

*The numbers in () are the numbers of metabolites enriched in the corresponding pathways.*

### Correlations Between the Milk Microbiome and Metabolites

**FIGURE 7:**
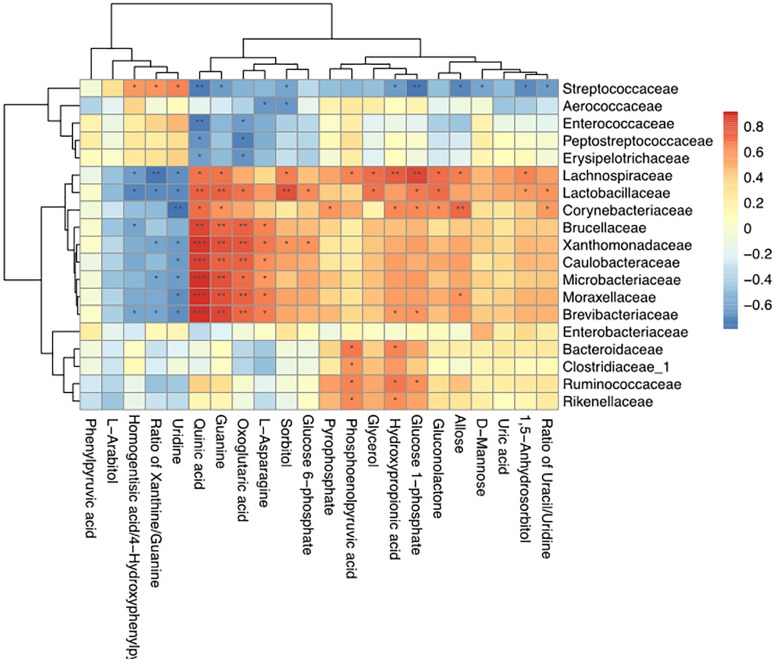
Correlation analyses between milk bacteria families and altered metabolite levels in healthy cows and those with *S. agalactiae* mastitis ^∗^0.01 < *p* < 0.05, ^∗∗^0.001 < *p* ≤ 0.01, ^∗∗∗^*p* ≤ 0.001.

Spearman correlation coefficients were calculated, and clear correlations were observed between the perturbed milk microbiome and the altered metabolite profiles (*r* > 0.5 or < −0.5, *p* < 0.05). Figure 7 shows that the significantly different types of metabolites between the groups were highly correlated with specific bacteria, demonstrating functional correlations between the milk microbiome and associated metabolites. We also found that *Streptococcaceae, Lachnospiraceae, Lactobacillaceae*, and *Corynebacteriaceae* were highly correlated with most metabolites. Only *Streptococcaceae* were significantly correlated with D-mannose levels. The most significantly changed metabolite ratio, the xanthine: guanine ratio, was positively correlated with *Streptococcaceae* and negatively correlated with *Lachnospiraceae, Lactobacillaceae, Xanthomonadaceae, Microbacteriaceae*, and *Brevibacteriaceae.*

## Discussion

In this study, we compared the milk microbiota diversity and metabolite profiles in CON dairy cows with those of cows with GBS. We used MiSeq high-throughput sequencing of 16S rRNA genes to profile the microbiota and GC-TOFMS for metabolite identification. Data on the milk microbiota not only revealed its richness and diversity but also demonstrated that its composition depended on the health and organ functionality of the animal (Weimer, 2014; Schokker et al., 2015; Junza et al., 2016). The observed metabolic changes provided further insight into the production of specific *S. agalactiae*-related metabolites and revealed that perturbations in milk metabolomic profiles can be used to explore differences in milk synthesis between CON cows and those with GBS.

### Multivariate Analysis of Milk Microbiomes of Healthy vs. Infected Cows

The microbial diversity in milk was dramatically decreased in the GBS group. Next-generation DNA sequencing has been widely used to evaluate microbial communities for in-depth characterization of the microbial profiles of specific ecosystems (Baltasar et al., 2014). Previous studies have indicated that there is a close relationship between reduced microbial diversity and mastitis in both human milk (Fernández et al., 2013) and bovine milk (Bhatt et al., 2012; Falentin et al., 2016). We found that the GBS group had a significantly lower Shannon index than the CON group (Table 3). These findings are consistent with those of previous studies indicating that there were more bacterial genera in non-infected quarters than in infected quarters (showing that diversity was related to health status; Braem et al., 2012) and that there was lower diversity in the uterine microbiota of cows suffering from metritis than in that of CON cows (Santos and Bicalho, 2012). However, several factors should be considered in accounting for the discrepancies between the groups, including the bacterial lysis and genomic DNA extraction methods, the different quarters sampled, etc. In a previous study, Henderson et al. (2013) clearly demonstrated that sampling techniques and DNA extraction methods can significantly affect the microbial compositions of cow and sheep rumens.

It has been shown that spoilage by *Acinetobacter* (Gurung et al., 2013), a serious problem in the dairy industry, as well as spoilage by *Streptococcus* (Zanardi et al., 2014), is strongly correlated with high SCCs (greatest size effect) as well as risk of GBS. Similar to previous studies that have described milk microbial communities, our study revealed the presence of *Acinetobacter* and *Streptococcus* in the milk samples (Figure 4). *Streptococcus thermophilus* generally produces bacteriocins that protect the final product from microbial spoilage (Kabuki et al., 2009). In our study, the bacterial population was extremely high in milk from cows with GBS, indicating that the metabolic end-products and enzymes of the bacteria had a considerable influence on milk quality.

One interesting observation from our study was the presence of *Firmicutes* and *Proteobacteria* in our milk samples (Figure 3). These species are common in the rumen microbiome (Tong et al., 2018; Wang et al., 2018), which may indicate a transfer between the rumen and the mammary gland, similar to the gut-breast axis observed in humans that allows transfer from mothers to neonates (Ted et al., 2014). Anne et al. (2010) reported that immune cells might be involved in the transport of microorganisms to different sites in the body, which may explain the high SCCs in milk from cows with GBS. Alternatively, the alterations in bacterial diversity may have resulted from fecal contamination through ascending colonization of the teat canal. In our study, we also found that *Lactobacillaceae* were highly negatively correlated with most metabolites associated with the GBS group (Figure 7). It has been reported that *Lactobacillus* lactic acid bacteria can degrade the disaccharide lactose, the major carbohydrate in milk (Huys et al., 2006). The proteolytic and lipolytic capacities of *Lactobacillus* in carbohydrate metabolism have considerable effects on the texture, taste, and aroma of fermented milk (Kim, 2014). These findings suggest that changes in the milk microbiota may be related to changes in milk yield or metabolic processes involved in milk synthesis.

### Differences in Milk Metabolites Between Healthy Dairy Cows and Those With Subclinical *S. agalactiae* Mastitis

Metabolomics, which integrates both technological and analytical approaches, can enable a comprehensive understanding of an organism’s physiological and biochemical status (Zhao et al., 2014). In this study, GC-TOFMS was used to compare metabolite levels and to determine differences in milk metabolites between healthy cows and those with GBS to discover potential biomarkers for milk quality responses to GBS. We identified 155 compounds in milk, more than previously identified in studies using nuclear magnetic resonance (NMR) (Hailemariam et al., 2014), liquid chromatography-mass spectrometry (LC-MS/MS) (Boudonck et al., 2009) and gas chromatography-mass spectrometry (GC–MS) approaches. The higher number of metabolites identified in this study shows that more sensitive detection and identification methods enable better qualitative identification of metabolites. Among the 22 significantly different metabolite parameters, five were more prevalent in the GBS group than in the control group, including phenylpyruvic acid, uridine, glycerol, the homogentisic acid: 4-hydroxyphenylpyruvic acid ratio, and the xanthine: guanine ratio (Table 4). It is known that aromatic compounds are derived from aromatic amino acids (Sébastien et al., 2012) such as tyrosine, which could account for the increases in phenylpyruvic acid (by 2.63-fold) and the homogentisic acid: 4-hydroxyphenylpyruvic acid ratio (by 2.92-fold) in the GBS group. Furthermore, phenylalanine is an essential amino acid and the precursor of catecholamines, which are neurotransmitters and adrenaline-like substances (Waisbren et al., 2007). Phenylalanine is transformed into tyrosine through the action of phenylalanine hydroxylase and a biopterin cofactor (Gilbert, 1992). Tyrosine is an important amino acid in many proteins, peptides, and even enkephalins and is also the precursor for hormones such as thyroxin and catecholoestrogens (Lemmon and Schlessinger, 2000). These characteristics may partially explain the variations in milk yield and quality between the groups.

It has previously been demonstrated that glycerol oxidation produces glyceric acid, which is mediated by the transfer of glucose carbon to serine in cows (Black et al., 1955). When glyceric acid is secreted excessively in milk, the lactating animal may suffer from D-glyceric aciduria and D-glycerate anemia, resulting in metabolic acidosis, progressive neurological impairment, seizures, hypotonia, and other adverse effects (JöRn Oliver et al., 2010). The current study revealed that glycerol was dramatically increased in the GBS group compared with the CON group, which could explain why GBS not only reduces milk yield and quality but also may lead to poor health. Thus, our data indicate that glycerol could be a potential biomarker for the diagnosis of GBS.

### Pathway Analysis of Healthy Dairy Cows vs. Those With Subclinical Mastitis

The current study not only identified differences in the metabolite profiles between the groups but also pinpointed the likely pathways that generated these metabolite differences. Based on an integrated analysis of the key metabolic pathways producing the 22 identified milk metabolites, carbohydrate metabolism appears to be the most important pathway associated with mastitis-induced metabolic changes in cows (Figure 6 and Table 5). It is well-known that carbohydrate metabolism performed by microorganisms in ruminant animals normally involves fatty acyl-CoA synthetases, which produce fatty acids, lactate and other products that can be absorbed in the intestine and transported to the mammary gland (Friggens et al., 1998). It has also been reported that alterations in the starch and sucrose pathway cause disorders of carbohydrate metabolism accompanied by significant increases in lactate and propionate and decreases in acetate (Ametaj et al., 2010; Pan et al., 2016). Moreover, since propionate is the predominant glucose precursor in ruminants (Ungerfeld, 2015), elevations in glucose stimulate milk protein synthesis (Rulquin et al., 2004). Interestingly, our correlation results revealed that the presence of *Streptococcaceae* was negatively correlated with the levels of D-mannose and glucose 1-phosphate (Figure 7). Thus, it is reasonable to conclude that *S. agalactiae* causes mammary gland metabolic dysfunction and milk composition changes. These data suggest a potential mechanism by which GBS affects milk composition.

In our pathway analysis, the TCA cycle was significantly stimulated in the GBS group compared to the CON group. The TCA cycle is the primary metabolic pathway in mitochondria and is critically important for the health of dairy cows. ATP is produced from a surplus of acetyl-CoA in the mitochondria (Wellen and Thompson, 2012) that originates from glucose, fatty acid, and lactate metabolism (Hardie and Carling, 2010). Previous studies have reported that microorganisms convert carbohydrates to pyruvate and acetyl-CoA through the glycolytic pathway and the pentose phosphate pathway (Chen L. et al., 2016). Due to the different needs of cells, ATP allocation is flexible. Pyrophosphate is a high-energy phosphate precursor that acts as a primary energy source for mitochondria in cells. Following creatinine formation, the pyrophosphate pathway plays an important role in monitoring energy status in tissues (Wyss and Kaddurahdaouk, 2000; Antunes-Fernandes et al., 2016). In our study, pyrophosphate levels were significantly lower in the milk of cows with GBS than in that of CON cows, which could have resulted in lower energy allocation to the mitochondria for milk synthesis in the infected cows (Table 4). Pyrophosphate and phosphoenolpyruvic acid were positively correlated with *Corynebacteriaceae* and with *Bacteroidaceae, Clostridiaceae, Ruminococcaceae*, and *Rikenellaceae*, respectively (Figure 7). Taken together, our results support the hypothesis that GBS alters the metabolite balance in cow milk by disrupting the TCA cycle in the mammary gland. This effect appears to be closely associated with milk yield and composition via changes in mitochondrial production of ATP as a fuel for metabolic activity.

In the present study, alanine, aspartate, and glutamate metabolism and arginine biosynthesis were significantly decreased in GBS cows compared to CON cows (Table 5). Previous reports have indicated that arginine, glycine, and methionine are the predominant amino acids for creatine synthesis in the liver (Poortmans et al., 2005). Creatine, an intermediate metabolite in energy reactions, plays an important role in regulating negative energy balance in dairy cows (Wang et al., 2016) and could also be a potential diagnostic biomarker for heat stress (Wang et al., 2016). Aspartate is a precursor for the biosynthesis of many essential compounds and participates in some cellular signaling pathways, such as gluconeogenesis, in dairy cows (Piccioli-Cappelli et al., 2014). Therefore, our results provide the potential molecular mechanisms underlying the observed differences in the cow milk metabolomes between the GBS and CON groups.

## Conclusion

Taken together, our data indicate that the structures of bacterial communities differ significantly between CON cows and those with GBS and that metabolic functions affecting milk composition are altered by GBS. 16S rRNA MiSeq high-throughput sequencing with metabolomic analysis revealed characteristic patterns. Our results also revealed comprehensive changes in essential metabolites associated with infection, as shown by the integrated pathway analyses, and suggest that some of the 22 different metabolites identified in the milk are potential diagnostic biomarkers for GBS. Through metabolomics, this study has provided insights into mechanisms that may partially explain changes in milk metabolites that occur in dairy cows upon infection with *S. agalactiae*, and our findings support further exploration of prophylactic strategies.

## Data Availability Statement

The datasets generated for this study can be found in the NCBI Sequence Read Archive (SRA; http://www.ncbi.nlm.nih.gov/Traces/sra/), under accession number SRP192494.

## Ethics Statement

The animal study was reviewed and approved by the Animal Care Committee, Beijing University of Agriculture (Beijing, China).

## Author Contributions

JT, HZ, BX, and LJ designed the study. JT, HZ, and YZ conducted the experiment. JT and HZ analyzed the data and wrote the manuscript. JT, HZ, LJ, and BX revised the manuscript. All authors carefully read the manuscript and agreed to be held accountable for all aspects of the work.

## Conflict of Interest

The authors declare that the research was conducted in the absence of any commercial or financial relationships that could be construed as a potential conflict of interest.

## Footnotes

1 https://international.biocloud.net/zh/user/login

2 www.metaboanalyst.ca/

3 http://www.ncbi.nlm.nih.gov/Traces/sra/
