# Supplementary material for: Microbiome and Metabolome Analyses of Milk From Dairy Cows With Subclinical Streptococcus agalactiae Mastitis—Potential Biomarkers
Source: Front Microbiol. 2019 Nov 6;10:2547. doi: 10.3389/fmicb.2019.02547 (PMC6851174; doi:10.3389/fmicb.2019.02547)
Supplement: Supplementary file 1 [file Table_1.docx]

Supplementary Table 1. Results of bacteriological culture and PCR of the milk samples

| Species | Number | Proportion* |
| --- | --- | --- |
| *Staphylococcus aureus* | 42 | 15.79% |
| *Streptococcus agalactiae* | 36 | 13.53% |
| *Escherichia coli* | 28 | 10.53% |
| *Coagulase-negative staphylococci* | 13 | 4.89% |
| *Streptococcus dysgalactiae* | 6 | 2.26% |
| *Staphylococcus aureus+Streptococcus* *agalactiae* | 3 | 1.13% |
| *Streptococcus uberis* | 1 | 0.38% |
| *Staphylococcus aureus+Streptococcus dysgalactiae* | 2 | 0.75% |
| *Pseudomonas aeruginosa* | 1 | 0.38% |
| *Klebsiella spp．* | 4 | 1.50% |
| *Bacillus spp．* | 2 | 0.75% |
| *Lactococcus lactis* | 4 | 1.50% |
| Not detected | 124 | 46.62% |
| Total | 266 | 100.00% |

*The proportion was calculated as the number of bacteria in each species or pair of species divided by the total number of bacteria.
